# Atypical hemolytic uremic syndrome (aHUS) responsive to mycophenolate mofetil: A case report from Nepal

**DOI:** 10.1016/j.amsu.2022.104932

**Published:** 2022-11-17

**Authors:** Jyoti Gurung, Anil Regmi, Bilash Poudel, Ritu Poudel, Rituraj Sharma, Sandip Raj Pradhan

**Affiliations:** a Civil Service Hospital, New Baneshwor, Kathmandu, Nepal; b Nepal Medical College Teaching Hospital, Kathmandu, Nepal; c Norvic International Hospital, Kathmandu, Nepal; d Nepal Mediciti Hospital, Lalitpur, Nepal; e All Nepal Hospital, Samakhusi, Kathmandu, Nepal

**Keywords:** Atypical hemolytic uremic syndrome, Management, Mycophenolate mofetil, Optic atrophy

## Abstract

**Introduction:**

An atypical hemolytic uremic syndrome is an extremely rare and life-threatening thrombotic microangiopathy. This disorder is caused by dysregulation of the alternative pathway of the complement system in association with genetic abnormalities or the development of autoantibodies. However, 30–50% of patients do not have genetic or acquired mutations in the complement system.

**Case report:**

Patient presented with fever and periorbital swelling. She had anemia, thrombocytopenia, and deranged liver function tests. Urinalysis revealed hematuria and proteinuria. Antibody tests and genetic analysis were negative. Renal biopsy revealed findings suggestive of thrombotic microangiopathy with predominantly glomerular involvement. Thus, the diagnosis of Atypical Hemolytic Uremic Syndrome, immunofluorescence negative, genetic negative, and anti-complement negative was made.

**Discussion:**

This article reports a case of atypical hemolytic uremic syndrome in a child with negative genetic analysis and anti-complement factor H antibody, which was treated successfully on steroid and mycophenolate mofetil. Early diagnosis along with prompt treatment and close monitoring will lead to recovery from atypical Hemolytic Uremic Syndrome.

**Conclusion:**

Although HUS is generally associated with genetic abnormalities or a positive antibody test, some patients with HUS may present atypically with negative genetic analysis and antibody tests.

## Highlights

• Hemolytic uremic syndrome (HUS) is a thrombotic microangiopathy.
• 5-10% of the cases of HUS is atypical HUS, mainly caused by abnormal activation of alternate complement pathway.
• Atypical hemolytic uremic syndrome results in a multi-organ system dysfunction, predominantly affecting the kidneys.
• Some patients with HUS may present atypically with negative genetic analysis and antibody tests.

## 1 Introduction

Hemolytic uremic syndrome (HUS) is a thrombotic microangiopathy (TMA) characterized by microangiopathic hemolytic anemia, thrombocytopenia, and renal impairment [1]. Typical hemolytic uremic syndrome is usually caused by Shiga toxin-producing bacteria *Escherichia coli* and Shigella dysentery [2]. Atypical hemolytic uremic syndrome (aHUS), which is mainly caused due to abnormal activation of the alternate pathway of complement activation, constitutes 5–10% of cases of HUS [3]. This uncontrolled complement activation is triggered by genetic abnormalities in complements of alternate pathways' most common complement factor H, which is an important regulatory protein, or due to the formation of autoantibodies against regulatory proteins, commonly CFH (anti-CFH autoantibodies). Abnormal and uncontrolled complement causes uncontrolled cleavage of the terminal complement protein complement 5 and excessive complement 5b-9 complex (membrane attack complex [MAC]) formation, which results in endothelial cell injury and activation leading to life-threatening TMA [3,4]. Atypical hemolytic uremic syndrome results in a multi-organ system dysfunction, predominantly affecting the kidneys [5]. We present a case of a 10-year-old female who presented with puffy eyes and decreased urine output at the age of 5 years and was later diagnosed with atypical hemolytic uremic syndrome. Clear-cut diagnostic criteria for aHUS have not been established to date. Diagnosis of aHUS involves ruling out other causes of TMA and incorporating complement serologic and genetic data [6]. Literature on aHUS in Nepal is very limited. This is the only reported case with recovery following a lengthy course of steroid and mycophenolate mofetil.

## 2 Case report

A five-year-old female child was brought to the pediatric department with complaints of fever, decreased urine output, periorbital swelling, and abdominal pain for three days. On evaluation, she had anemia (Hb = 8.4 g/dl) and thrombocytopenia (17,000/mm3). Her serum albumin was 3.1g/dl, serum creatinine was 0.6 mg/dl, and serum urea was 22 mg/dl. Her liver function test showed elevated SGOT (303 U/L) and SGPT (117 U/L). She had plenty of RBCs on urine routine analysis with proteinuria (urine protein 3+, urine protein-to-creatinine ratio 2.45). She was ANA negative, Anti-ds DNA negative, DCT negative, and had a Complement C3 level of 142 mg/dl (normal range 75–135mg/dl). The child was initially diagnosed with a case of autoimmune hemolytic anemia. She was managed with platelet-rich plasma transfusion, steroids, and other supportive care. Bone marrow aspiration was done, which showed normal hematopoiesis. She was discharged after a month of hospitalization when her blood and urine parameters returned to normal.

The child was well till seven years of age when she presented with fever for three days, decreased urine output for one day, along with the passage of cola-colored urine. The symptoms associated were headache, vomiting, and periorbital swelling. On evaluation, she had anemia (lowest hemoglobin 7.4 g/dl), thrombocytopenia (platelet count 75,000/mm3), highest corrected reticulocyte count 7.3%, schistocyte index 4.8, peak low-density lipoprotein (LDL) 396 mg/dl. Her urine routine examination revealed plenty of RBCs, her urine protein was 4+, and 24-h urine protein excretion was 1.26 g. Acute kidney injury (AKI) was seen with a rapid rise in creatinine within 3 days (serum creatinine raised from 1.8mg/dl to 4.8mg/dl). Her Complement C3 level was 76, peak serum urea was 276 mg/dl, SGOT was 323 IU/L, SGPT was 250 IU/L, and DCT was negative. Systolic blood pressure ranged from 120 to 140 mm Hg, and diastolic blood pressure ranged from 80 to 100 mm Hg.

With the possibility of Rapidly Progressive Glomerulonephritis (RPGN), the child was given four pulses of methylprednisolone and oral cyclophosphamide (for approximately seven days). During her hospital stay, she had 1 episode of seizure. She complained of blurring of vision; the fundoscopy revealed bilateral optic atrophy as a sequela of ischemic retinopathy. Ultrasound of abdomen during hospital stay revealed slightly increased bilateral renal echotexture with Grade I medical-renal disease. A renal Biopsy was done during the hospital stay, revealing findings suggestive of thrombotic microangiopathy with predominantly glomerular involvement with the onset of chronicity. Immunofluorescence was negative.

After reviewing the renal biopsy report, atypical HUS was suspected, and genetic tests were done. Genetic analysis for deletion/duplication variations in CHF, CFHR1, and CFHR3 genes was negative. In addition, an anti-complement factor H Antibody analysis was done, which showed negative results.

The diagnosis of Atypical Hemolytic Uremic Syndrome, immunofluorescence negative, genetic negative, and anti-complement negative was made. The child was started on diuretics, anti-hypertensives, anticonvulsant (phenytoin) for seizures, and oral prednisolone. Her condition improved after one month of hospital stay. On discharge, her renal function test was within the normal range. She was discharged on the above medications.

The child was kept under close monitoring. On her monthly follow-up, her urine report revealed trace albumin and 10–15 RBCs, though gross hematuria had resolved. The dose of anti-hypertensive was decreased, and the steroid was planned to be tapered over six months. Oral mycophenolate mofetil was started. Blood pressure monitoring was encouraged, and the child was kept in a close follow-up. Over the following months, her eye symptoms gradually improved. By six months, her blood pressure was within the normal range. She had no episode of seizure in this period. Her renal function test was normal, and her complete blood count had improved. Urine routine examination and 24-h protein-to-creatinine ratio were also normal. Thus, steroids, anti-hypertensives, and phenytoin were stopped, and mycophenolate mofetil was continued. Subsequent follow-up showed improving laboratory parameters, which meant that she was in remission. Hence, mycophenolate mofetil stopped after two years. The patient is currently not on any medication and has no symptoms.

## 3 Discussion

The patient represents a typical case of atypical hemolytic uremic syndrome with a severe form of this disease. She developed several complications, such as acute kidney injury, refractory hypertension, seizure, and optic atrophy. This case proves that early diagnosis along with prompt treatment and close monitoring will lead to recovery from aHUS.

The patient's initial clinical presentation indicated AKI due to glomerulonephritis, including massive proteinuria, hematuria, and azotemia. Furthermore, RPGN was suspected because of the rapid deterioration of renal function. The occurrence of AKI, microangiopathic hemolytic anemia, and thrombocytopenia represented the clinical triad that fulfilled the diagnostic criteria for TMA [7]. Renal biopsy revealed findings suggestive of thrombotic microangiopathy with predominantly glomerular involvement. Genetic analysis for deletion/duplication variations in CHF, CFHR1, and CFHR3 genes was negative. Anti-complement factor H Antibody analysis was done, which showed negative results. Long-term treatment with corticosteroids and mycophenolate mofetil, along with various supportive therapies, led to a drastic improvement in her kidney function and other symptoms.

The main aim during the acute phase of treatment of aHUS is to remove the circulating autoantibodies, which is achieved by early initiation of plasma exchange and administration of corticosteroids and immunosuppressants (cyclophosphamide or rituximab or mycophenolate mofetil) to reduce or prevent continued autoantibody production [8]. This therapy is followed by maintenance of immunosuppression by starting immunosuppressant treatment like mycophenolate mofetil [9]. Immunosuppressive drugs such as MMF have shown long-term dialysis-free survival in 60–70% of patients [9]. A monoclonal antibody, Eculizumab, acts by blocking the terminal complement pathway. It can also be used as an alternative to an immunosuppressive regimen during the maintenance phase [10,11]. In our case, immunosuppression with mycophenolate mofetil for two years led to clinical improvement in the child.

## 4 Conclusion

Atypical HUS is a rare disease associated with high morbidity and mortality. Early diagnosis and close monitoring will help significant recovery from the illness. Due to the recent advances, many treatment options are available depending on the stage of the disease. Treatment modalities include plasma exchange, eculizumab, immunosuppressants, and/or dialysis in the end stages. This case report has been written in line with the SCARE 2020 criteria [12].

## Provenance and peer review

Not commissioned, externally peer-reviewed.

## Ethical consideration

According to the local ethical guideline, ethical approval is not necessary to write a case report. We obtained written consent from the patient to include the clinical details including pictures.

## Ethical approval

According to the local ethical guideline, it is not mandatory for ethical approval for writing a case report. Written informed consent was obtained from the patient's parents to include the clinical details.

## Sources of funding

No funding was available for this research.

## Author contribution

Jyoti Gurung, Anil Regmi, Bilash Poudel, Ritu Poudel, Rituraj Sharma, and Sandip Raj Pradhan: reviewed the literature and designed the manuscript.

Jyoti Gurung, Anil Regmi, and Ritu Poudel: established the diagnosis, coordinated the patient's management with Pediatrician and Nephrologists, and treated the patient.

All authors read and approved the final version of the manuscript.

## Registration of research studies

1. Name of the registry: N/A
2. Unique Identifying number or registration ID: N/A
3. Hyperlink to your specific registration (must be publicly accessible and will be checked): N/A

## Guarantor

Dr. Anil Regmi, Department of Internal Medicine, Nepal Medical College Teaching Hospital, Kathmandu, 44,600, Nepal. Email: aregmi45new@gmail.com Phone: +977–9849071817 ORCID ID: 0000-0003-0896-8107.

## Consent

Informed consent for the possible publication of this case report was taken from the patient's parents and consent form was signed by father.

## Declaration of competing interest

No authors have any conflict of interest.
